# Low diversity and abundance of predatory fishes in a peripheral coral reef ecosystem

**DOI:** 10.1002/ece3.10920

**Published:** 2024-02-09

**Authors:** Collin T. Williams, Francesco Garzon, Jesse E. M. Cochran, Lyndsey K. Tanabe, Lucy A. Hawkes, Ashlie J. McIvor, Ameer A. Eweida, Paul A. Marshall, Michael L. Berumen

**Affiliations:** ^1^ Red Sea Research Center, Division of Biological and Environmental Science and Engineering King Abdullah University of Science and Technology Thuwal Saudi Arabia; ^2^ Hatherly Laboratories University of Exeter, Biosciences, Faculty of Health and Life Sciences Exeter UK; ^3^ MARE—Marine and Environmental Sciences Centre/ARNET‐Aquatic Research Network Regional Agency for the Development of Research, Technology and Innovation (ARDITI) Funchal Madeira Portugal; ^4^ NEOM—Marine and Environmental Division Riyadh Saudi Arabia; ^5^ NEOM Nature Reserve NEOM Riyadh Saudi Arabia; ^6^ James Cook University Townsville Queensland Australia

**Keywords:** biogeography, fisheries, Gulf of Aqaba, marginal seas, Red Sea, sharks

## Abstract

Semi‐enclosed seas are often associated with elevated local threats and distinct biogeographic patterns among marine fishes, but our understanding of how fish assemblage dynamics vary in relation to relatively small semi‐enclosed seas (e.g., the Gulf of Aqaba) remains limited. Baited remote underwater video surveys (*n* = 111) were conducted across ~300 km of coral reef habitats in the Gulf of Aqaba and the northern Red Sea. A total of 55 predatory fish species were detected, with less than half of all species (*n* = 23) observed in both basins. Relative abundance patterns between the Gulf of Aqaba and the northern Red Sea were variable among taxa, but nearly twice as many predatory fish were observed per unit of effort in the northern Red Sea. In general, assemblages in both basins were dominated by three taxa (Epinephelinae, Carangidae, and Lethrinidae). Large‐bodied and threatened species were recorded at very low abundances. Multivariate analysis revealed distinct assemblage structuring of coral reef predators between the Gulf of Aqaba and the northern Red Sea. Most of the species driving these differences were recorded in both basins, but occurred at varying levels of abundance. Environmental factors were largely unsuccessful in explaining variation in assemblage structuring. These findings indicate that biological assemblages in the Gulf of Aqaba are more distinct than previously reported and that reef fish assemblage structuring can occur even within a relatively small semi‐enclosed sea. Despite inter‐basin assemblage structuring, the overall low abundance of vulnerable fish species is suggestive of overexploitation in both the Gulf of Aqaba and the northern Red Sea of Saudi Arabia. As the region surveyed is currently undergoing large‐scale coastal development, the results presented herein aim to guide spatial management and recovery plans for these coral reef systems in relation to this development.

## Graphical abstract

Our findings provide insights into the structuring of coral reef fish communities, demonstrating that distinct assemblages can occur within semi‐enclosed seas at smaller spatial scales than previously documented. We also report a low abundance of threatened fish species in an area of rapid, large‐scale coastal development, which identifies an urgent need to develop a regional management plan.

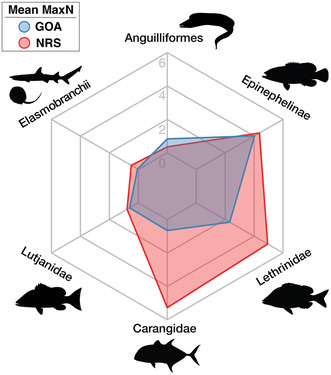

## 1 INTRODUCTION

Predatory fishes are a key component of both coral reef ecosystems and tropical fisheries. Predators serve important roles in the functioning of reef systems by shaping the abundance, composition, and behavior of prey species as well as redistributing nutrients among habitats (Almany, 2004; Roff et al., 2016; White et al., 2010; Williams et al., 2018). Removal of reef predators has been associated with pronounced community‐level shifts and other ecological changes, indicating their broader functional importance on reefs (Boaden & Kingsford, 2015; Dulvy et al., 2004; Hammerschlag et al., 2018; Rasher et al., 2017). In addition, predatory fishes often constitute a significant portion of the catch from coastal fisheries in tropical regions (Munro, 1996). As such, reef predators are also vital to both food security and local economies in many tropical nations (McManus, 1996). Despite strong ecological and economic justifications for maintaining healthy populations of predatory reef fish, substantial declines, especially among sharks and rays, have been documented worldwide primarily as a result of overexploitation (Dulvy et al., 2021; MacNeil et al., 2020; Simpfendorfer et al., 2023; Wilson et al., 2008).

Understanding how and why fish assemblages vary spatially can inform tailored approaches to the management of coral reef fisheries. For instance, determining areas of elevated fish diversity, abundance, and biomass allows for ecologically significant locations to be prioritized for protection (Jenkins & Van Houtan, 2016). Identifying the variables that influence reef fish assemblages (e.g., temperature, salinity, and shelf position: Khalil et al., 2017; Mellin et al., 2010) can further support management efforts by enabling spatial predictions of biological patterns. However, more complex factors, such as fishing pressure, benthic habitat features, and evolutionary histories can also influence the structuring of reef fish assemblages and result in deviations from broadly recognized patterns at varying spatial scales (Eisele et al., 2021; Hall & Kingsford, 2021; Kiflawi et al., 2006). Accordingly, data at fine spatial scales can be crucial to establishing successful regional management plans for reef fisheries (Nash & Graham, 2016), especially in heterogeneous habitats, where ecological barriers can occur over relatively short distances.

Semi‐enclosed seas are relatively isolated bodies of water, some of which are known to contain unique regions of biodiversity and climate refugia (Hodge et al., 2012; Johannesson & André, 2006; Osman et al., 2018). The geographic structure of these basins (i.e., small basins almost entirely surrounded by land) also renders semi‐enclosed seas particularly susceptible to anthropogenic influences (Caddy, 1993, 2000), and pronounced declines of predatory fishes have been documented within their boundaries (Buchanan et al., 2019; Sala et al., 2004; Spaet et al., 2016; Vasilakopoulos et al., 2014). Environmental barriers to the dispersal of marine species are generally associated with semi‐enclosed seas (DiBattista et al., 2016; Nihoul, 1982; Ormond & Roberts, 1997), so it is unsurprising that many large semi‐enclosed seas (1000–2000 km) harbor fish communities with a distinct species composition [e.g., Red Sea (DiBattista et al., 2016), Arabian Sea (Burt et al., 2011), and Gulf of California (Robertson & Cramer, 2009)]. This pattern does not appear to persist in the Gulf of Aqaba (Khalaf & Kochzius, 2002), a similarly structured but much smaller basin (~200 km). However, published community‐level comparisons of reef fish assemblages in the Gulf of Aqaba are largely based on the presence and absence of species (Khalaf & Kochzius, 2002; Roberts et al., 1992) and it remains unclear whether these data provide adequate resolution to assess the distinctiveness of fish assemblages in this basin.

The primary objective of this study was to compare diversity and abundance patterns of predatory coral reef fishes in the peripheral marine ecosystem of the Gulf of Aqaba with fish assemblages from the northern Red Sea. A narrow channel of water (the Straits of Tiran) serves as the only connection between the Gulf of Aqaba and the main basin of Red Sea, providing an ideal context to explore if and how spatial patterns of reef fish assemblages vary in relation to apparent physical barriers at a spatial scale much smaller than typically associated with ecoregions and biogeographical realms (Costello et al., 2017; Spalding et al., 2007). Additionally, a considerable portion of the Gulf of Aqaba and the northern Red Sea coastline (~460 km) lies within NEOM (http://neom.com), a large‐scale development project in Saudi Arabia, which is set to drive substantial change in land use and coastal infrastructure in the region. Thus, we aim to provide both a novel examination of reef fish biogeography and inform conservation planning in an area of rapid coastal development.

## 2 METHODS

**FIGURE 1:**
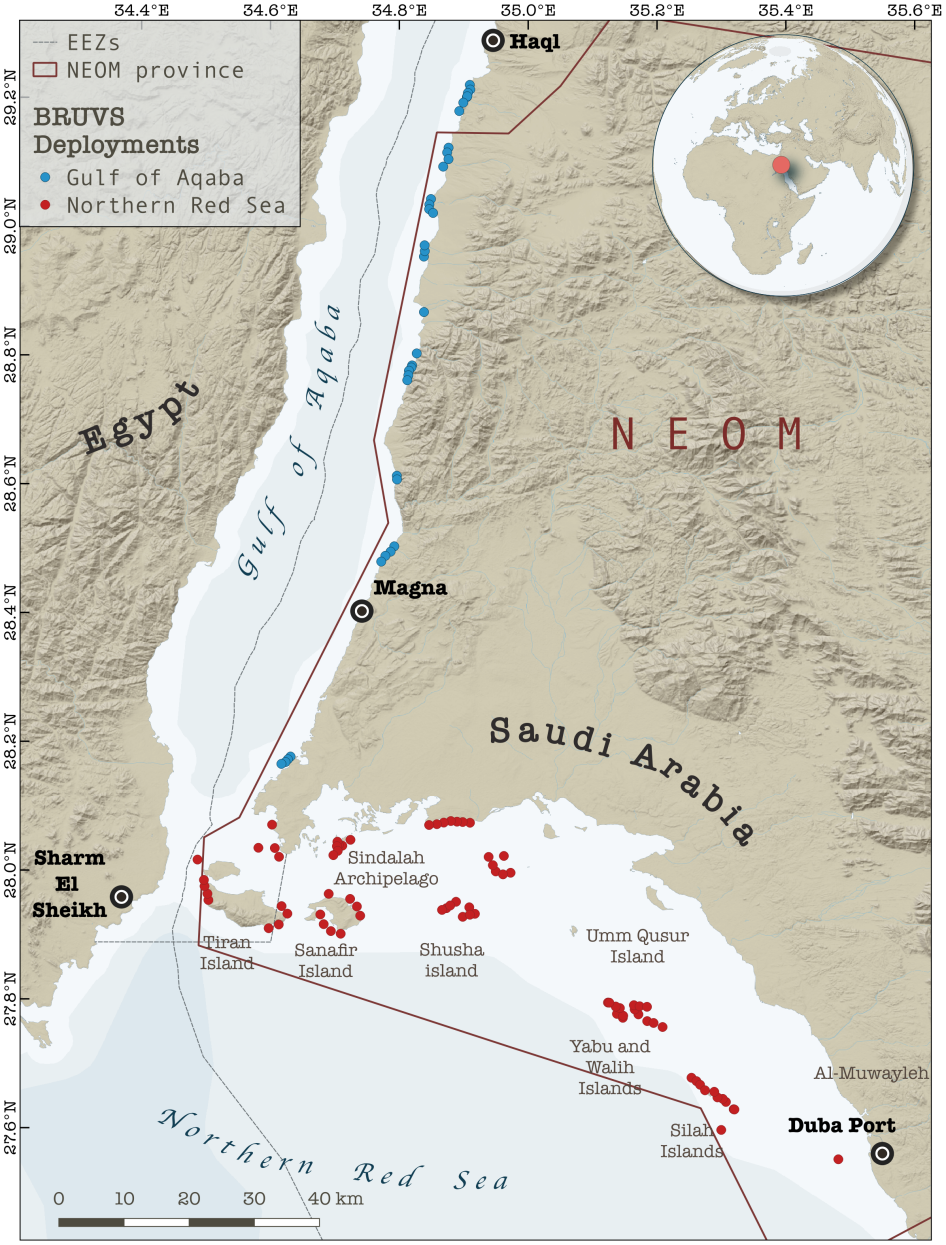
Map of the Gulf of Aqaba and the northern Red Sea denoting the location of baited remote underwater video system (BRUVS) deployments (blue and red dots). Dashed black lines represent the limits of the exclusive economic zones (EEZs) of the countries bordering the Red Sea; continuous red line delineates the boundaries of the NEOM province. Basemap layer created in MapBox software (https://www.mapbox.com) using data from the Natural Earth repository (https://www.naturalearthdata.com).

Baited remote underwater video systems (BRUVS), a widely used and standardized survey method (see MacNeil et al., 2020; Simpfendorfer et al., 2023), were used to characterize predatory fish assemblages on coral reefs in the Gulf of Aqaba and the northern Red Sea. The northern Red Sea is defined in this study as the main basin of the Red Sea north of 27° latitude, excluding the Gulf of Aqaba and the Gulf of Suez. The southern extent of the Gulf of Aqaba was delineated at 28.1° N latitude corresponding with the easternmost point of Ras Al‐Sheikh Hamid. Deployments were conducted along ~300 km of coastal environments predominantly within the territory of NEOM (Figure 1). Survey methods followed protocols for BRUVS deployment outlined by Global Finprint (https://globalfinprint.org) and further described in MacNeil et al. (2020). The standard Global Finprint metal BRUVS frame was used for surveys, fitted with a 1.5 m bait arm and GoPro HERO6 camera. Each deployment contained ~1 kg of bait cut into small chunks [either kawakawa (*Euthynnus affinis*) or frigate tuna (*Auxis* sp.) obtained from markets along the Saudi Arabian Red Sea coastline]. BRUVS were deployed on the seafloor during daylight hours, targeting a depth of approximately 20 m, and left to record for at least 65 minutes. To avoid interference, synchronously deployed BRUVS were placed at least 400 m apart. The seafloor depth at the deployment site was measured using the boat's onboard sensors, or a HawkEye H22PX Handheld Depth Finder. Time of BRUVS deployment, BRUVS retrieval, and GPS coordinates were recorded for each site.

The first 5 min of footage following BRUVS settlement on the seafloor was discarded, and only the subsequent 60 min of footage was analyzed. Two experienced researchers with knowledge of regional ichthyofauna independently reviewed footage from each BRUVS deployment. Reviewers recorded predatory fishes to the lowest taxonomic level possible, the time of appearance, and the maximum number of individuals present in a single video frame (MaxN). Predatory fishes in the following taxa were recorded: subclass Elasmobranchii (sharks and rays), the order Anguilliformes (eels), and the (sub)families: Carangidae (jacks), Epinephelinae (groupers; excluding cryptic, small‐bodied species from the tribe Grammistini), Labridae (only the large‐bodied wrasse *Cheilinus undulatus*), Lethrinidae (emperors), Lutjanidae (snappers), Scombridae (mackerels and tunas), and Sphyraenidae (barracudas). Due to complexities in visual discrimination, observations of two emperor species (*Lethrinus microdon* and *L. olivaceus*) were combined and dark‐spotted whipray individuals (*Himantura* spp.) were identified as *Himantura uarnak* based on recent genetic evidence from the Red Sea (Borsa et al., 2021). Following initial video processing, reviewers met to reconcile any discrepancies between observations.

A limited number of environmental variables were available for subsequent analysis, including in‐water visibility, habitat type, distance to mainland (m), and mean annual sea surface temperature (SST, see below), which were documented for each deployment. In‐water visibility of video footage was visually ranked on a scale of 1–4 (from best to worst; Figure S1). Deployments assigned a visibility rank of 4 (i.e., extremely poor visibility) were excluded from analysis. Habitat types were assigned to one of three groups corresponding to coral cover and structure: coral‐dominated reef crests, coral‐dominated expansive platforms, and sand‐dominated benthos surrounding small (i.e., <500 m) patch reefs and coral bombies (referred to as H1, H2, and H3, respectively; see Figure S2). Distance to mainland, defined as the distance (in meters) from BRUVS coordinates to the nearest large continental land mass (excluding islands), was calculated for each deployment in QGIS (v 3.16.4) using 1:10 m coastline data obtained from Natural Earth (https://www.naturalearthdata.com/). Mean annual SST for each site in 2020 was calculated using NOAA High Resolution SST data (extracted at 0.25° × 0.25° resolution) provided by the NOAA/OAR/ESRL PSL, Boulder, Colorado, USA (https://psl.noaa.gov).

All statistical analyses were conducted using the software R v 4.0.5 (R Core Team, 2021), and maps were created in QGIS. Calculations of species accumulation curves, diversity indices, and multivariate comparisons were conducted in R through the *vegan* package (Oksanen et al., 2020). The ability of BRUVS deployments to capture the total number of predator species was assessed using species accumulation curves, where a curve approaching an asymptote was taken to infer that the majority of species had been observed in the surveyed regions. Standardized species richness values (*S′*) were obtained through randomized bootstrapping of 1000 permutations. For comparisons of abundance, total MaxN was calculated as the sum of MaxN values for all species in a given predatory fish grouping (i.e., basins or taxonomic groups). From this, an effort‐standardized measure of abundance, mean MaxN, was then obtained by dividing total MaxN by the number of BRUVS deployments in the corresponding grouping (total MaxN/1 h of BRUVS effort). Relative abundance (RA) was calculated by dividing the total MaxN of a taxonomic group (i.e., species or family) by the total MaxN of all deployments and species in a given basin. The Shannon–Wiener diversity index was derived using the following equation: $H^{\prime }=-\sum_{i=1}^{S}p_{i}\;\ln\;p_{i}$, where *S* is species richness and *p*_*i*_ is the RA of a species *i*. Pielou's *J* of species evenness was calculated as follows: $J^{\prime }=\frac{H^{\prime }}{\log\left(S\right)}$. Statistical comparisons of abundance and diversity between predatory fish assemblages in the Gulf of Aqaba and the northern Red Sea were explored using a two‐sample Mann–Whitney *U* test for mean MaxN values and one‐way ANOVAs for *H′* and *J'* values among deployments.

Multivariate visualization and analysis were employed to make further comparisons of predator assemblages between basins, to explore the deterministic role of environmental variables, and to identify the species driving any potential differences in assemblage structuring. To scale species abundances, data were normalized among BRUVS deployments by constraining the sum of squares to one. The assumption of homogeneous multivariate dispersion was evaluated with a permutational distance‐based test PERMDISP (Anderson, 2006). Differences between predator assemblages in the northern Red Sea and in the Gulf of Aqaba were evaluated with the permutational multivariate analysis of variance (PERMANOVA) (Anderson, 2017). Data were depicted in multivariate space with non‐metric multidimensional scaling (NMDS) using Bray–Curtis distances (Bray & Curtis, 1957). The percent contribution of specific species to the overall Bray–Curtis dissimilarity between the Gulf of Aqaba and the northern Red Sea was determined using a similarity percentage routine (Clarke, 1993). Variation in predator assemblage structure driven by environmental variables was quantified and visualized by distance‐based multivariate multiple regression and distance‐based redundancy analysis, respectively.

## 3 RESULTS

**FIGURE 2:**
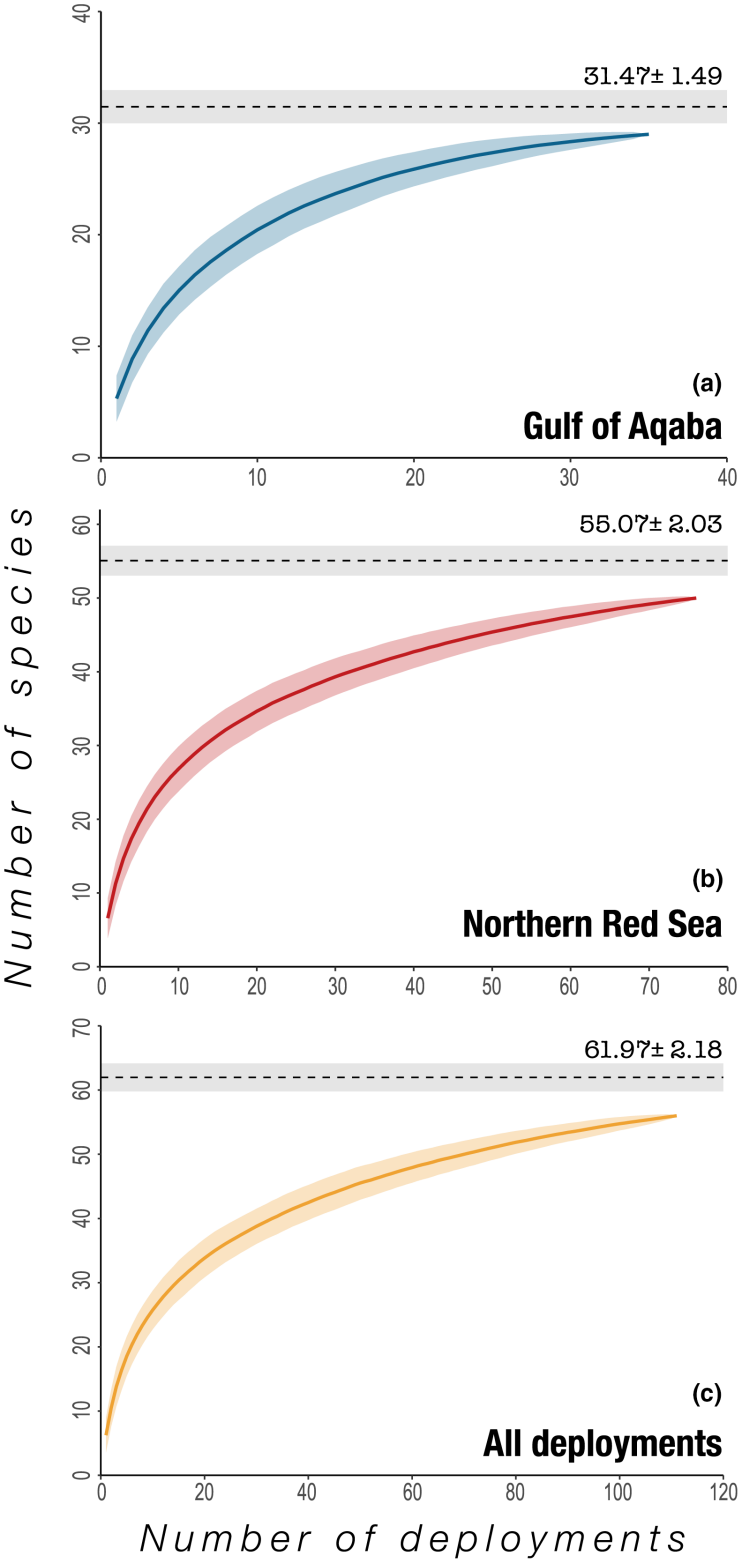
Species accumulation curves for BRUVS deployments (a) within the Gulf of Aqaba, (b) within the northern Red Sea and (c) within both areas combined. Note x‐ and y‐axis scales vary between plots. Colored shading indicates standard error around the estimated curves. Dashed line and gray shading indicate the predicted asymptotic total number of species in each region and the associated standard error, respectively.

A total of 111 BRUVS deployments were conducted over a period of 29 days (October 13–November 10, 2020) at an average depth of 18.8 m ± 0.5 SE (Table 1, Table S1). A consensus of MaxN and species identity was reached between reviewers for all observations. Across all deployments, 55 species were detected, of which 26 species (47%) were recorded exclusively in the northern Red Sea and six species (11%) were recorded exclusively in the Gulf of Aqaba (Tables 1 and 2). Less than half of all species detected (*n* = 23 species, 42%) were found in both the Gulf of Aqaba and the northern Red Sea (Tables 1 and 2). Species accumulation curves suggested that adequate sampling effort had been conducted (Figure 2). Bootstrapping furthermore indicated a higher species richness (*S′*) in the northern Red Sea than in the Gulf of Aqaba, with 49 species recorded in the northern Red Sea out of the 55 species predicted to occur in the basin and 29 species recorded in the Gulf of Aqaba out of the 31 species predicted (Table 1, Figure 2). Species evenness (*J'*) was significantly higher in the Gulf of Aqaba (ANOVA: *p* = .03, *F*_(1,109)_ = 5.13), corresponding to a lower number of rare species (i.e., species that were infrequently detected). No differences in Shannon–Wiener diversity index values (*H′*) were found between the basins (ANOVA: *p* = .24, *F*_(1,109)_ = 1.43).

**TABLE 1:** Deployment quantities, diversity metrics, and abundance measures of predatory fishes obtained from baited remote underwater video systems (BRUVS) in the Gulf of Aqaba (GOA) and the northern Red Sea (NRS).

Deployments		Diversity				Abundance	
Region	BRUVS (*n*)	*S*	*S′*	*H′*	*J'*	Total MaxN	Mean MaxN
GOA	35	29	31.5 ± 1.5	2.772	0.823	309	8.8
NRS	76	49	55.1 ± 2.0	2.799	0.719	1266	16.9
Total	111	55	62.0 ± 2.2	2.898	0.723	1575	14.1

Abbreviations: *H′*, Shannon–Wiener diversity index; *J'*, Pielou's Evenness; Mean MaxN, the mean number of individuals observed per hour of survey effort; *S*, observed species richness; *S′*, species richness derived from bootstrapping; Total MaxN, the total number of individuals recorded.

**TABLE 2:** Metrics of abundance and prevalence for predatory fishes detected with BRUVS in the Gulf of Aqaba (GOA) and the northern Red Sea (NRS), including the total number of individuals recorded (Total MaxN), the greatest number of individuals record in a single deployment (Max MaxnN), and the average number of indivuals recorded among deployments (Mean MaxN).

Broad taxonomic classification	Species	Total MaxN	Max MaxN	Mean MaxN (total)	Mean MaxN (GOA)	Mean MaxN (NRS)	NOcc	FO (%)	RA (%)
Anguilliformes		56	3	0.5	0.83	0.36	44	39.64	3.56
	*Gymnothorax flavimarginatus*	4	2	0.04	0.11	—	2	1.8	0.25
	*Gymnothorax griseus*	4	1	0.04	0.09	0.01	4	3.6	0.25
	*Gymnothorax javanicus*	30	2	0.27	0.14	0.33	29	26.13	1.9
	*Gymnothorax nudivomer*	14	2	0.13	0.40	—	12	10.81	0.89
	*Gymnothorax undulatus*	3	1	0.03	0.06	0.01	3	2.7	0.19
	*Myrichthys maculosus*	1	1	0.01	0.03	—	1	0.9	0.06
Carangidae		424	143	3.82	0.66	5.28	68	61.26	26.92
	*Atule mate*	15	11	0.14	—	0.2	3	2.7	0.95
	*Carangoides bajad*	303	143	2.73	0.26	3.87	53	47.75	19.24
	*Carangoides ferdau*	3	2	0.03	—	0.04	2	1.8	0.19
	*Carangoides fulvoguttatus*	15	2	0.14	0.09	0.16	13	11.71	0.95
	*Carangoides plagiotaenia*	9	4	0.08	—	0.12	4	3.6	0.57
	*Caranx ignobilis*	2	1	0.02	—	0.03	2	1.8	0.13
	*Caranx melampygus*	47	7	0.42	0.31	0.47	24	21.62	2.98
	*Scomberoides lysan*	29	17	0.26	—	0.38	2	1.8	1.84
	*Seriolina nigrofasciata*	1	1	0.01	—	0.01	1	0.9	0.06
Elasmobranchii		39	4	0.35	0.06	0.49	33	29.73	2.48
Sharks		21	2	0.19	0.03	0.26	20	0.18	1.33
	*Carcharhinus amblyrhynchos*	2	1	0.02	—	0.03	2	1.8	0.13
	*Carcharhinus brevipinna*	1	1	0.01	—	0.01	1	0.9	0.06
	*Carcharhinus melanopterus*	3	1	0.03	—	0.04	3	2.7	0.19
	*Carcharhinus plumbeus*	1	1	0.01	0.03	—	1	0.9	0.06
	*Nebrius ferrugineus*	1	1	0.01	—	0.01	1	0.9	0.06
	*Triaenodon obesus*	13	1	0.12	—	0.17	13	11.71	0.83
Rays		18	3	0.16	0.03	0.22	15	0.14	1.14
	*Aetobatus ocellatus*	4	2	0.04	—	0.05	3	2.7	0.25
	*Himantura uarnak*	1	1	0.01	—	0.01	1	0.9	0.06
	*Pastinachus sephen*	2	1	0.02	—	0.03	2	1.8	0.13
	*Taeniura lymma*	7	1	0.06	—	0.09	7	6.31	0.44
	*Taeniurops meyeni*	1	1	0.01	0.03	—	1	0.9	0.06
	*Torpedo panthera*	1	1	0.01	—	0.01	1	0.9	0.06
	*Urogymnus granulatus*	2	1	0.02	—	0.03	2	1.8	0.13
Epinephelinae		473	13	4.26	4.03	4.37	103	92.79	30.03
	*Aethaloperca rogaa*	15	2	0.14	0.09	0.16	13	11.71	0.95
	*Anyperodon leucogrammicus*	2	1	0.02	—	0.03	2	1.8	0.13
	*Cephalopholis argus*	48	5	0.43	0.11	0.58	27	24.32	3.05
	*Cephalopholis hemistiktos*	162	5	1.46	1.14	1.61	80	72.07	10.29
	*Cephalopholis miniata*	59	8	0.53	0.83	0.39	30	27.03	3.75
	*Epinephelus fasciatus*	55	5	0.5	0.69	0.41	30	27.03	3.49
	*Epinephelus fuscoguttatus*	2	2	0.02	—	0.03	1	0.9	0.13
	*Epinephelus malabaricus*	5	1	0.05	—	0.07	5	4.5	0.32
	*Plectropomus areolatus*	5	1	0.05	—	0.07	5	4.5	0.32
	*Plectropomus marisrubri*	35	2	0.32	0.09	0.42	28	25.23	2.22
	*Variola louti*	85	3	0.77	1.09	0.62	66	59.46	5.4
Labridae	*Cheilinus undulatus*	14	2	0.13	0.06	0.16	13	11.71	0.89
Lethrinidae		456	34	4.11	2.31	4.93	73	65.77	28.95
	*Gymnocranius grandoculis*	8	2	0.07	0.09	0.07	7	6.31	0.51
	*Lethrinus borbonicus*	298	29	2.68	1.49	3.24	52	46.85	18.92
	*Lethrinus mahsena*	29	3	0.26	0.34	0.22	22	19.82	1.84
	*Lethrinus microdon*	64	18	0.58	0.2	0.75	19	17.12	4.06
	*Lethrinus nebulosus*	16	5	0.14	0.14	0.14	8	7.21	1.02
	*Lethrinus variegatus*	5	5	0.05	—	0.07	1	0.9	0.32
	*Lethrinus xanthochilus*	19	3	0.17	0.06	0.22	14	12.61261	1.21
	*Monotaxis grandoculis*	17	6	0.15	—	0.22	8	7.21	1.08
Lutjanidae		80	5	0.72	0.6	0.78	44	39.64	5.08
	*Lutjanus bohar*	66	5	0.59	0.54	0.62	39	35.14	4.19
	*Lutjanus monostigma*	1	1	0.01	—	0.01	1	0.9	0.06
	*Macolor niger*	11	2	0.1	—	0.14	9	8.11	0.7
	*Paracaesio sordida*	2	2	0.02	0.06	—	1	0.9	0.13
Scombridae		28	4	0.25	0.23	0.26	21	18.92	1.78
	*Grammatorcynus bilineatus*	26	4	0.23	0.23	0.24	20	18.02	1.65
	*Gymnosarda unicolor*	2	1	0.02	—	0.03	2	1.8	0.13
Sphyraenidae	*Sphyraena barracuda*	5	1	0.05	0.06	0.04	5	4.5	0.32

*Note*: The number of occurrences (NOcc) corresponds to the total number of BRUVS in which the species occurred, the frequency of occurrence (FO) equals the NOcc divided by the total number of BRUVS deployed, and relative abundance (RA) indicates percent composition relative to the entire survey effort. Note that MaxN values associated with broader taxonomic groups represent the sum of the MaxNs for each individual species within a single BRUVS deployment, and not MaxN for the group within a single video frame.

**FIGURE 3:**
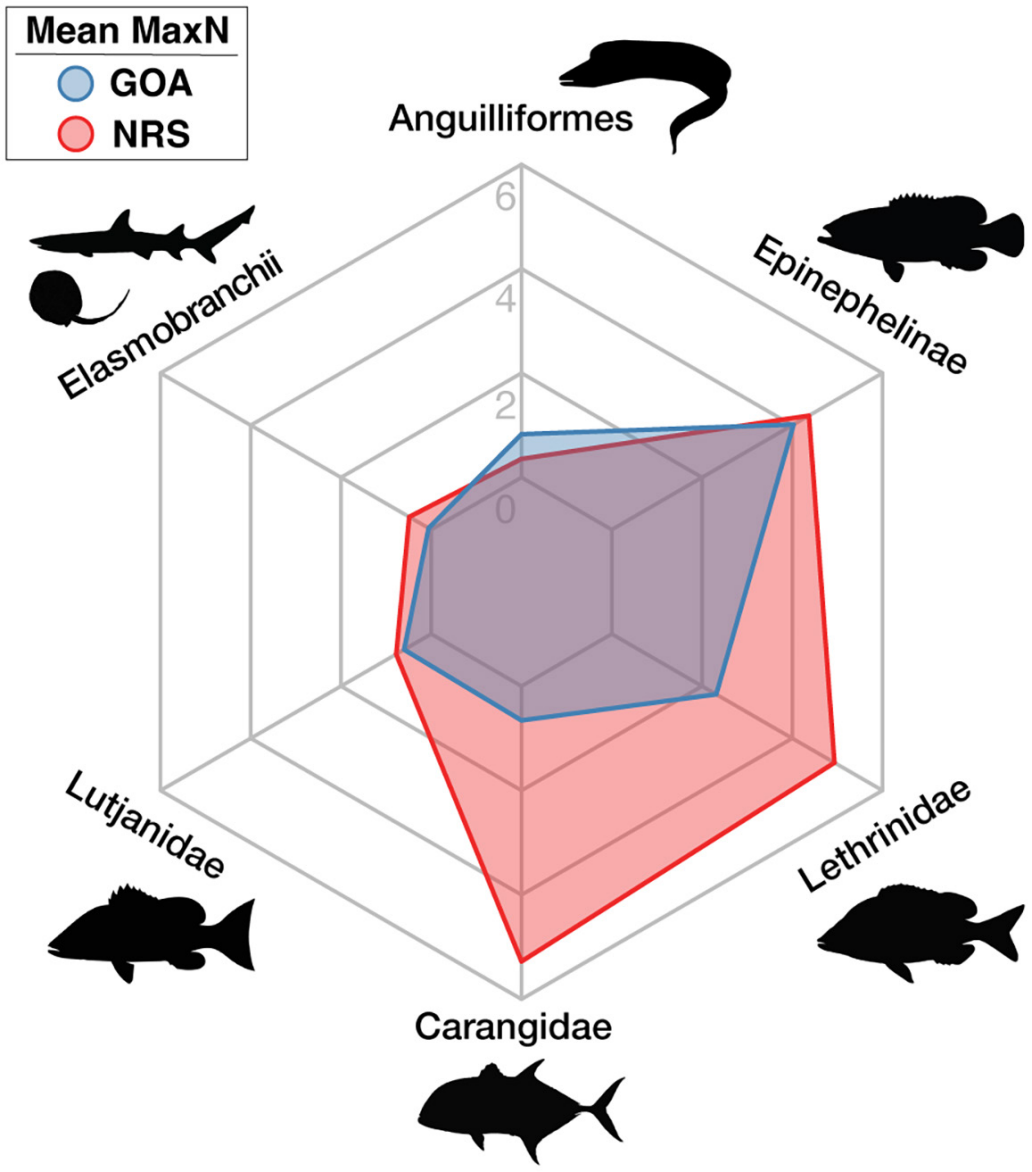
Spider plot depicting the mean MaxN of the most abundant coral reef predatory fish taxa (overall mean MaxN >0.25) in the Gulf of Aqaba (GOA) and the northern Red Sea (NRS). Silhouettes from Phylopic (http://phylopic.org) and the fishualize package in R (Schiettekatte et al., 2019).

**FIGURE 4:**
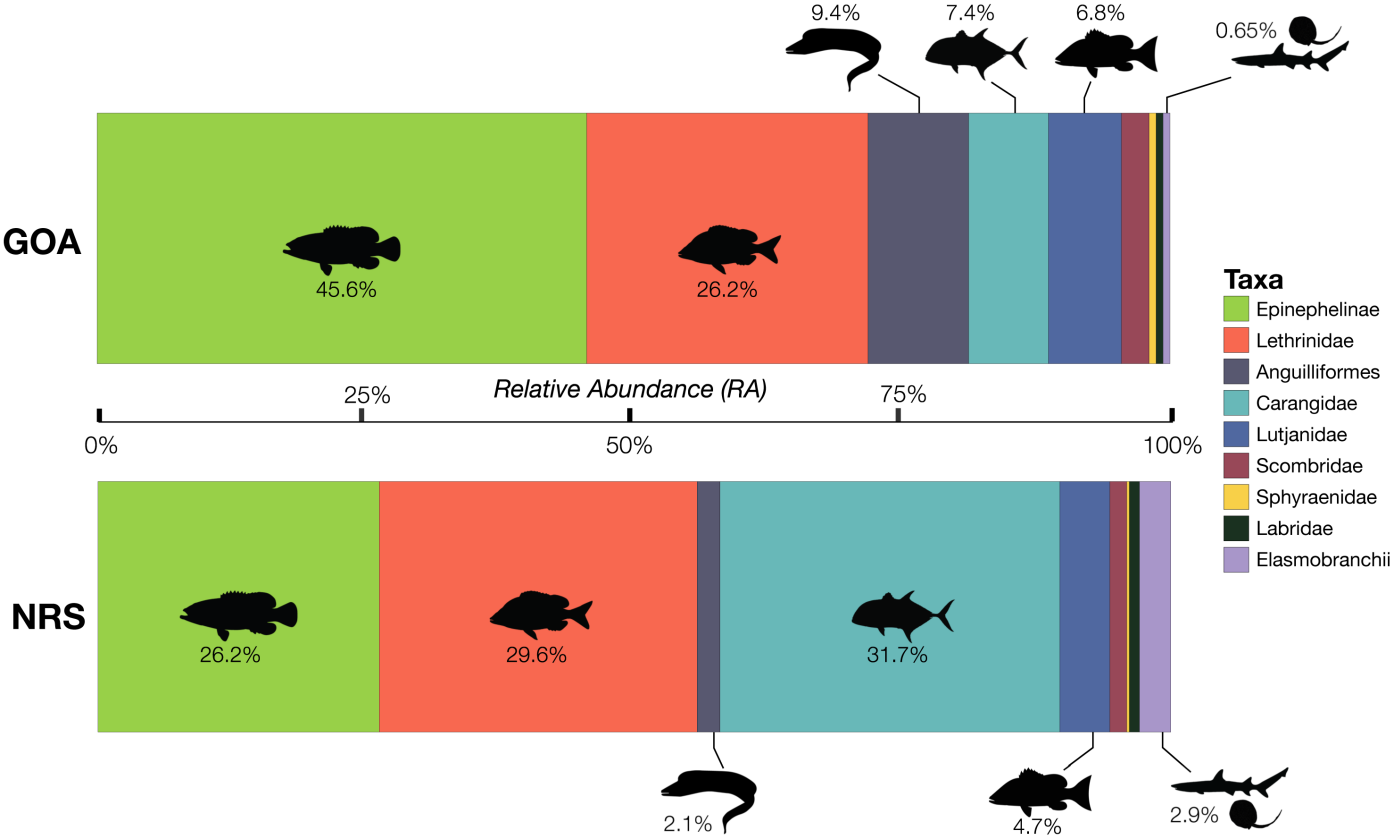
Relative abundance of coral reef predatory fishes in the Gulf of Aqaba (GOA) and the northern Red Sea (NRS). The horizontal lengths of each section of each bar represent the proportion of each taxon group in each basin. Note that the number of individuals recorded in each basin are not equal (GOA = 309 individuals, NRS = 1266 individuals). Silhouettes from Phylopic (http://phylopic.org) and the fishualize package in R (Schiettekatte et al., 2019).

**FIGURE 5:**
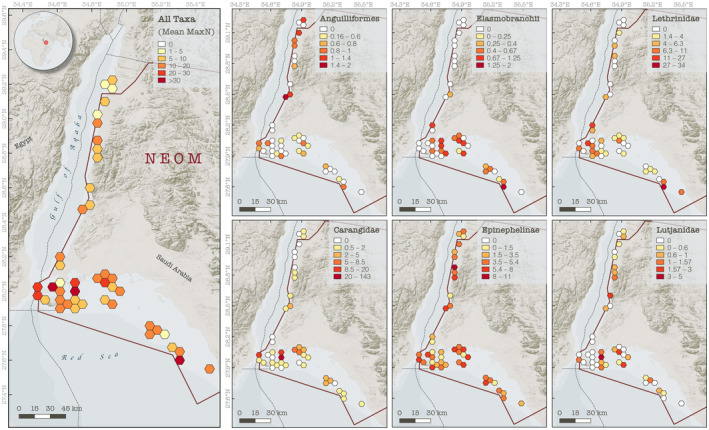
Distribution of mean MaxN for the most abundant coral reef predatory fish taxa in the study area. MaxN values represent the mean of all BRUVS deployments within a hexagonal grid cell. Note that the color scale (indicated in the legends) varies for each group, and the number of deployment within each is also variable. Dashed lines represent the Exclusive Economic Zones of the countries bordering the Red Sea; continuous red line delineates the border of the NEOM project. Basemap layer created in MapBox software (https://www.mapbox.com) using data from the Natural Earth repository (https://www.naturalearthdata.com).

Significant differences in the overall abundance of coral reef predators (i.e., mean MaxN of all species combined) were observed between basins (Mann–Whitney *U* test: *p* = .001, W = 825) with approximately twice as many predatory fish recorded per unit effort in the northern Red Sea compared to the Gulf of Aqaba (mean MaxN of 16.9 in the northern Red Sea versus 8.8 in the Gulf of Aqaba, Table 1). With the exception of eels and barracudas, all surveyed taxa were more abundant in the northern Red Sea than in the Gulf of Aqaba (Figure 3, Table 2). Three key predatory fish taxa dominated assemblages in the northern Red Sea [Carangidae (RA = 31.7%), Epinephelinae (RA = 26.2%), and Lethrinidae (RA = 29.6%)], whereas carangids made up a much smaller proportion of the Gulf of Aqaba assemblage [Carangidae (RA = 7.4%), Epinephelinae (RA = 45.6.2%), and Lethrinidae (RA = 26.2%)] (Figure 4, Table 2). Overall, a low abundance of sharks and rays was recorded, with relatively greater diversity and abundance found in the northern Red Sea (Figure 3, Table 2). Large‐bodied (i.e., species that reach ≥2.5 m TL), mobile predators were observed at low abundances in surveys, consisting of just four individual sharks [*Carcharhinus plumbeus* (*n* = 1), *Carcharhinus amblyrhynchos* (*n* = 2), and *Carcharhinus brevipinna* (*n* = 1)] (Table 2). Fine‐scale spatial trends among all BRUVS deployments suggest areas of elevated predator abundance in close proximity to the Straits of Tiran, Sindalah Archipelago, and offshore reef pinnacles near Sila Island, although such patterns were not observed among all taxonomic groups (Figure 5).

**FIGURE 6:**
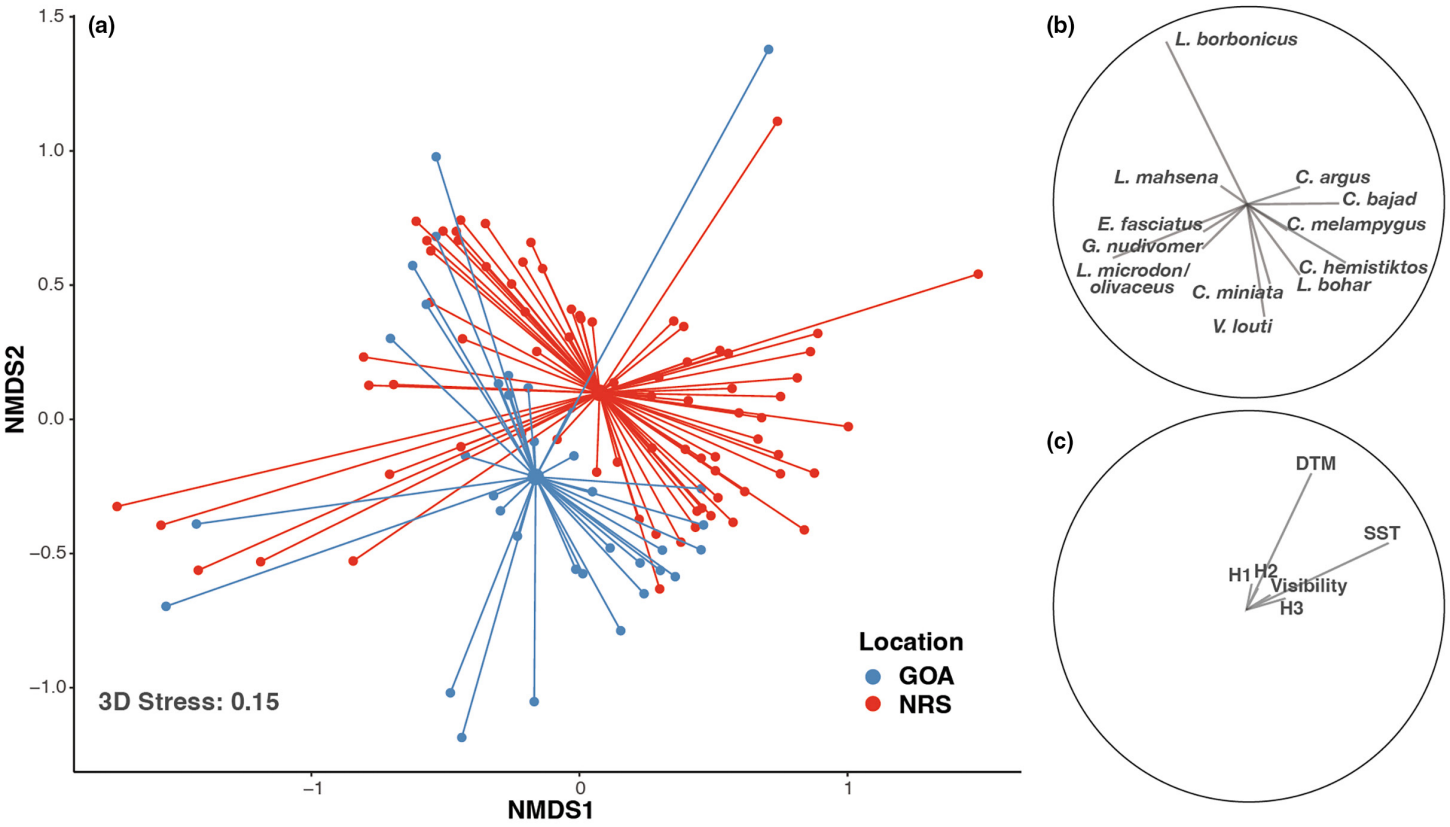
Multivariate analyses of BRUVS deployments from the Gulf of Aqaba (GOA) and the northern Red Sea (NRS), including: (a) Non‐metric multidimensional scaling using Bray–Curtis distances in which each small point represents a single deployment and color denotes basin (larger points portray the centroid of each basin with lines radiating out to respective deployments), (b) Pearson correlation vectors of species responsible for ≥3% dissimilarity between basins, and (c) environmental variables vectors visualized using distance‐based redundancy analysis (visibility, distance to mainland (DTM), sea surface temperature (SST), and three habitat types (H1–H3); described in detail in Methods and Figure S2). Vectors represent the strength and direction of corresponding relationships.

Multivariate analysis revealed differences in the overall assemblage structure of coral reef predators between the Gulf of Aqaba and the northern Red Sea (PERMANOVA: *p* < .001, *F*_(1,109)_ = 4.23). The magnitude of variation within assemblages was similar between basins (PERMDISP: *p* = .723, *F*_(1,109)_ = 0.15) enabling multivariate comparisons. NMDS illustrated distinctly clustered but overlapping predator assemblages, one corresponding to the Gulf of Aqaba and the other to the northern Red Sea, with wide variability observed in both basins (Figure 6). Twelve species were responsible for over 70% of the dissimilarity in assemblage structure between the Gulf of Aqaba and the northern Red Sea (Table 3; Figure 6). While 11 of these 12 species were detected in both basins, many exhibited considerable variation in abundance between the Gulf of Aqaba and the northern Red Sea (e.g., *Lethrinus borbonicus*, *Carangoides bajad*, and *Gymnothorax nudivome*r), likely driving the distinct structuring between assemblages (Table 3; Figure 6). Two environmental variables were found to have a significant effect on assemblage structuring, distance to mainland (*p* = .002, *F*_(1,103)_ = 3.08) and mean annual SST (*p* < .001, *F*_(1,103)_ = 4.41), but these variables accounted for a small portion of differences between the Gulf of Aqaba and the northern Red Sea (2.7% and 3.0%, respectively; Table 4). Most of the variation in assemblage structuring between the Gulf of Aqaba and the northern Red Sea (90.4%) could not be explained by the variables considered (Table 4).

**TABLE 3:** Percent contribution of the most influential species to the overall Bray–Curtis dissimilarity of predatory fish assemblages between the Gulf of Aqaba (GOA) and the northern Red Sea (NRS) determined using a similarity percentage routine.

Species	Sub(family)	% dissimilarity contribution	% cumulative contribution	GOA mean MaxN	NRS mean MaxN	GOA RA (%)	NRS RA (%)
*Lethrinus borbonicus*	Lethrinidae	12.0	12.0	1.5	**3.2**	16.8	**19.4**
*Cephalopholis hemistiktos*	Epinephelinae	9.6	21.5	1.1	**1.6**	**12.9**	9.6
*Variola louti*	Epinephelinae	7.6	29.1	**1.1**	0.6	**12.3**	3.7
*Carangoides bajad*	Carangidae	6.4	35.5	0.3	**3.9**	2.9	**23.2**
*Cephalopholis miniata*	Epinephelinae	6.2	41.7	**0.8**	0.4	**9.4**	2.4
*Epinephelus fasciatus*	Epinephelinae	5.9	47.6	**0.7**	0.4	**7.8**	2.4
*Lutjanus bohar*	Lutjanidae	5.4	53.0	0.5	**0.6**	**6.1**	3.7
*Lethrinus microdon/olivaceus*	Lethrinidae	4.3	57.3	0.2	**0.8**	2.3	**4.5**
*Cephalopholis argus*	Epinephelinae	3.6	61.0	0.1	**0.6**	1.3	**3.5**
*Lethrinus mahsena*	Lethrinidae	3.6	64.6	**0.3**	0.2	**3.9**	1.3
*Caranx melampygus*	Carangidae	3.5	68.1	0.3	**0.5**	**3.6**	2.8
*Gymnothorax nudivomer*	Anguilliformes	3.0	71.2	**0.4**	—	**4.5**	0

*Note*: Mean MaxN and percent relative abundance (RA) values of each species from both regions are additionally provided (greatest value between basins denoted in bold).

**TABLE 4:** Significance and percent explained variation of environmental factors on the assemblage structure of predatory fishes between the Gulf of Aqaba and the northern Red Sea.

Variable	*p*	% explained variation
DML	**.002**	2.7
Visibility	.788	0.6
Habitat	.585	2.4
Mean Annual SST	**<.001**	3.9
Residual	—	90.4

*Note*: The examined environmental variables include, distance to mainland (DML), visibility (Figure S1), habitat type (Figure S2), and mean annual sea surface temperature (SST). Reported statistics are derived from distance‐based multivariate multiple regression. Significant variables (*p* < .05) are denoted in bold.

## 4 DISCUSSION

Despite the considerable volume of marine biological research conducted in the Gulf of Aqaba (Berumen et al., 2013), there has been no consensus in the literature as to whether or not the basin is biologically or ecologically distinct from the Red Sea's main basin (Berumen et al., 2019; Carvalho et al., 2018; DiBattista et al., 2016). The results of this study demonstrate that biological assemblages within the Gulf of Aqaba can be distinct, further indicating that the unique structuring of reef fish assemblages often observed within semi‐enclosed seas can also occur within relatively small systems. Our findings provide insights into the spatial structuring of fish communities on coral reefs and suggest that management strategies should consider the distinctiveness of peripheral environments at smaller spatial scales. Regardless of the differences observed between basins, the overall low predatory fish abundance throughout the entire surveyed area indicates regional declines that warrant wide‐ranging conservation actions across the Gulf of Aqaba and the northern Red Sea.

Unlike larger semi‐enclosed seas (e.g., Arabian Gulf a Gulf of California), the borders of which correspond to boundaries of species distributions (DiBattista et al., 2016; Robertson & Cramer, 2009; Spalding et al., 2007), most of the species recorded from the Gulf of Aqaba in this study occur widely throughout the region (Golani & Fricke, 2018). The Gulf of Aqaba does, however, host several species that are thought to be endemic, though this has been questioned, with studies suggesting that these species have simply yet to be documented elsewhere (DiBattista et al., 2016). This speculation is supported by the basin's relatively small size, gene flow with the Red Sea's main basin (DiBattista et al., 2020; Froukh & Kochzius, 2007; Kochzius & Blohm, 2005), and presence of many fish species in both the Gulf of Aqaba and the Red Sea (Khalaf & Kochzius, 2002). Irrespective of species distributions, spatial variations of species abundance can correspond to distinct structuring of biological assemblages (Khalil et al., 2017). Yet, the few studies which have examined reef fish abundance in the region have not offered explicit, detailed comparisons between the Gulf of Aqaba and the northern Red Sea, often focusing on a limited number of species and overlooking larger predatory fishes (Alwany, 2008; Roberts et al., 1992; Saleh et al., 2019; Sawall et al., 2014). Our findings confirm that fish assemblages in the Gulf of Aqaba are more distinct than previously reported, highlighting the value of incorporating abundance estimates into comparisons of reef fauna over smaller spatial scales.

Most of the variance between predatory reef fish assemblages in the Gulf of Aqaba and the northern Red Sea is driven by species that were observed in both basins, but at very different abundances. In most cases, the predatory taxa targeted in this study tended to be more abundant in the northern Red Sea compared to the Gulf of Aqaba. This was especially pronounced in some species (e.g., *L. borbonicus*, *Cephalopholis hemistoktos*, *C. bajad*) including those Red Sea species that have still never been recorded in the Gulf of Aqaba, such as the mangrove whipray (*Urogymnus granulatus*) and barcheek trevally (*Carangoides plagiotaenia*) (Golani & Fricke, 2018). Few species have demonstrated a relatively greater prevalence in the Gulf of Aqaba (e.g., *Tylosarus choram* [El‐Dawi, 2000], *Cephalopholis miniata*), with the most striking example being the yellow‐mouth moray (*G. nudivomer*). This eel species is widely distributed throughout the Indo‐Pacific, but was not detected in the northern Red Sea despite being present at over 30% of BRUVS deployments in the Gulf of Aqaba. Through examining abundance patterns spatially, it becomes clear that many other species of predatory fishes have patchy local distributions, often exhibiting uneven abundances between the Gulf of Aqaba and the northern Red Sea. This underscores the significance of considering not only the presence of a species but also their relative abundance, when characterizing coral reef fish assemblages spatially. Understanding the specific species that drive these differences can provide further insights into the processes that shape the composition and structure fish communities on coral reefs.

The distinct patterns of predatory fish assembly observed between the Gulf of Aqaba and the northern Red Sea may be attributed to varying environmental factors. Relative to the Red Sea's main basin, the Gulf of Aqaba exhibits lower water temperatures and higher salinity (Reiss & Hottinger, 1984). Temperature in particular played a significant role in explaining assemblage‐level differences between basins in this study, but the mechanisms driving these assemblage differences remain unclear. One explanation is that species‐specific physiological constraints influences the recruitment success and overall fitness of certain fishes, resulting in distinctly structured assemblages. There is also a possibility that the thermal niche of certain species occurs at different depths in each basin. If so, our sampling, which targeted a specific depth, may have underreported or not documented certain species. Another potential explanatory factor is the differing geomorphological structure of the continental shelf in this region. The Gulf of Aqaba is characterized by a single continuous fringing reef along an extremely steep continental shelf that drops to depths of ~2000 m just ~2 km from shore, whereas the more gradually sloped shelf of the northern Red Sea spans ~20 km with a complex network of islands and reefs (Ribot et al., 2021). These stark differences in shelf structure likely correspond to variations in the availability of shallow coral reef habitat and the carrying capacity of these systems, which may be associated with the greater abundance of predatory fishes in the northern Red Sea relative to the Gulf of Aqaba. The shift in diversity and composition of predatory fishes between these basins could also be a result of distinct shelf‐associated habitat features that were not captured by our coarse characterization of habitats. In the future, coupling reef fish assemblage surveys with in situ environmental data and detailed benthic characterizations may yield greater insights into the environmental factors associated with the assemblage patterns described in this study.

Human activities have also likely played a role in shaping coral reef predator assemblages in the Gulf of Aqaba and the northern Red Sea. There appear to be almost no enforced fishing regulations throughout the entire survey area, although geopolitical sensitivity surrounding the Straits of Tiran and restricted access to large sections of the Saudi Arabian northern Red Sea may function partially as de facto marine protected areas. The elevated abundance of predatory fish surrounding the Straits of Tiran could also be influenced by spillover from established marine protected areas nearby in Egypt (Ashworth & Ormond, 2005). In contrast, the single fringing reef along a steep shelf in the Gulf of Aqaba exposes the entirety of its shallow reef habitat to shore‐based fishing pressure. Considerable amounts of fishing line cover large sections of reef in the Gulf of Aqaba near Haql, Saudi Arabia (Figure S3; authors' personal observations), which may be indicative of spatially concentrated fishing efforts along the coast of this basin. Regional fishing pressure on coral reefs has been associated with lower predator abundance, absence of sensitive species (e.g., sharks), and altered assemblage structuring (Kattan et al., 2017), which is consistent with relative predator assemblage patterns we observed in the Gulf of Aqaba. A similar, but even more pronounced lack of predatory and commercially harvested fishes has also been reported along Jordanian Gulf of Aqaba coast, where no carcharhinid, lutjanid, or scombrid was observed in BRUVS surveys (Attum et al., 2023). The higher abundance of eels in the Gulf of Aqaba may serve as an additional indication of overfishing and top predator removal, as inverse relationships between eel and shark abundance have been reported elsewhere (Clementi et al., 2021). In addition, a large area of coral reef in the Gulf of Aqaba between Ras Suwayhil al Saghir and Magna, Saudi Arabia was smothered in rocky debris resulting from coastal blasting for road construction. Although undocumented, it is apparent that this disturbance has impacted the underlying benthic community, potentially leading to shifts in predator assemblages due to subsequent changes in prey availability. The findings presented here do suggest that reef fish communities in the Gulf of Aqaba have experienced a relatively greater level of anthropogenic influence compared to the northern Red Sea.

Although predatory fish assemblages on shallow reefs in the Gulf of Aqaba and northern Red Sea are clearly distinct, both basins exhibit characteristics of overfishing, especially among large‐bodied and sensitive species. This is particularly evident for *C. undulatus*, a large wrasse species currently listed as Endangered (Russell, 2004). The low abundance of *C. undulatus* in this study is consistent with fished areas in other regions and is approximately three to 10 times less abundant than in protected areas with enforcement (Birt et al., 2021). Sharks and rays are also particularly vulnerable to fishing pressure in coral reef systems (MacNeil et al., 2020; Simpfendorfer et al., 2023), and previous BRUVS surveys in the northern Red Sea of Saudi Arabia did not document any sharks and only an extremely low abundance of rays (mean MaxN = 0.02). While we found a greater diversity and abundance of sharks and rays than previously recorded in the region, we also surveyed different habitats, and the abundance values reported in this study are still indicative of population declines associated with fished areas (Simpfendorfer et al., 2023; Spaet et al., 2016; Speed et al., 2018). Although natural variations in reef shark abundance do occur among regions, BRUVS‐derived shark depletion indices still point to a near total depletion of blacktip (*Carcharhinus melanopterus*), whitetip (*Triaenodon obesus*), and gray reef shark (*C. amblyrhynchos*) populations throughout the NEOM area (Simpfendorfer et al., 2023). Collectively, our findings support the need to enact fishing regulations and protections for predatory reef fishes along Saudi Arabia's coastline of the Gulf of Aqaba and the northern Red Sea. Quantifying current anthropogenic pressures on these coastal ecosystems will be an important next step towards determining the variables influencing coral reef fish assemblages in this region and establishing corresponding conservation measures.

Disentangling the environmental and anthropogenic factors shaping predatory fish assemblages was beyond the scope of the current study, but we were able to provide meaningful baseline information for the spatial management of reef fauna in the Gulf of Aqaba and the northern Red Sea. Observed differences in predatory fish communities indicate the possible utility of establishing separate conservation priorities for the Gulf of Aqaba and the northern Red Sea. For instance, in the Gulf of Aqaba, priority may be given to establishing and enforcing coastal harvest restrictions throughout the basin, while in the northern Red Sea, priority could be placed upon protecting known areas with relatively high diversity and abundance of predatory fishes. However, it is important that the description of biological differences between these two basins does not overshadow the pressing need for protections of predatory fishes across the entire region as indicated by the low abundance of vulnerable fished species. Our findings further highlight considerable conservation potential at the Straits of Tiran, the Sindala Archipelago, and the Sila reef pinnacles, some of which have previously been identified as potential priority areas for conservation (Garzon et al., 2022; Gladstone et al., 2003). Effectively protecting these areas may be particularly critical as the Gulf of Aqaba and the northern Red Sea are expected to serve as a climate refuge for coral reef ecosystems (Cacciapaglia & van Woesik, 2016; Osman et al., 2018). As the NEOM development project progresses and human populations increase regionally, the management of this marine environment will also become increasingly more complex. While the findings of this study provide a vital foundation for ongoing spatial planning, continued monitoring as well as surveys at different depths and habitats (e.g., Asher et al., 2017; McIvor et al., 2022) will be necessary for making informed decisions to conserve reef fishes in the Gulf of Aqaba and the northern Red Sea.

## AUTHOR CONTRIBUTIONS

**Collin T. Williams:** Conceptualization (equal); data curation (equal); formal analysis (equal); investigation (equal); methodology (equal); project administration (equal); software (equal); validation (equal); visualization (equal); writing – original draft (lead); writing – review and editing (equal). **Francesco Garzon:** Conceptualization (equal); data curation (equal); formal analysis (equal); investigation (equal); methodology (equal); project administration (equal); software (equal); validation (equal); visualization (equal); writing – review and editing (equal). **Jesse E. M. Cochran:** Conceptualization (equal); data curation (equal); investigation (equal); methodology (equal); validation (equal); writing – review and editing (equal). **Lyndsey K. Tanabe:** Data curation (equal); investigation (equal); validation (equal); writing – review and editing (equal). **Lucy A. Hawkes:** Conceptualization (equal); data curation (equal); investigation (equal); methodology (equal); project administration (equal); supervision (equal); writing – review and editing (equal). **Ashlie J. McIvor:** Formal analysis (equal); software (equal); visualization (equal); writing – review and editing (equal). **Ameer A. Eweida:** Funding acquisition (equal); resources (equal); writing – review and editing (equal). **Paul A. Marshall:** Funding acquisition (equal); resources (equal); writing – review and editing (equal). **Michael L. Berumen:** Conceptualization (equal); funding acquisition (equal); methodology (equal); project administration (equal); resources (equal); supervision (equal); writing – review and editing (equal).

## FUNDING INFORMATION

This research was supported by KAUST (baseline research funds to M.L.B.) and the NEOM Company (2020 NEOM‐OceanX Red Sea Expedition).

## Supporting information


Appendix 1.

## Data Availability

The data supporting these findings are available in Table 2 and the appendices of this article.
