# Supplementary material for: Low diversity and abundance of predatory fishes in a peripheral coral reef ecosystem
Source: Ecol Evol. 2024 Feb 9;14(2):e10920. doi: 10.1002/ece3.10920 (PMC10857893; doi:10.1002/ece3.10920)
Supplement: Supplementary file 1 — Appendix 1. [file ECE3-14-e10920-s001.docx]

**Appendix for Manuscript:**

**Low diversity and abundance of predatory fishes in a peripheral coral reef ecosystem**

Collin T. Williams^1^, Francesco Garzon^2^, Jesse E. M. Cochran^1^, Lyndsey K. Tanabe^1^, Lucy A. Hawkes^2^, Ashlie J. McIvor^1,3^, Ameer A. Eweida^4^, Paul A. Marshall^5,6^, Michael L. Berumen^1^

^1^ Red Sea Research Center, Division of Biological and Environmental Science and Engineering, King Abdullah University of Science and Technology, Thuwal, Kingdom of Saudi Arabia 23955-6900

^2^ Hatherly Laboratories, University of Exeter, Biosciences, Faculty of Health and Life Sciences, Exeter, EX4 4PS, UK

^3^ MARE - Marine and Environmental Sciences Centre/ ARNET-Aquatic Research Network, Regional Agency for the Development of Research, Technology and Innovation (ARDITI), Funchal, Madeira, Portugal

^4^ NEOM - Marine and Environmental Division, Riyadh, Kingdom of Saudi Arabia

^5^ NEOM Nature Reserve, NEOM, Kingdom of Saudi Arabia

^6^ James Cook University, Townsville, Australia

**Correspondence:** Collin Williams

Email: collin.williams@kaust.edu.sa

Figure S1: Four categories of visibility assigned to deployments in the Gulf of Aqaba and the northern Red Sea. Increasing rank number corresponds to increasingly impaired visibility, with a rank of 1 denoting excellent water clarity and a rank of 4 denoting poor visibility.


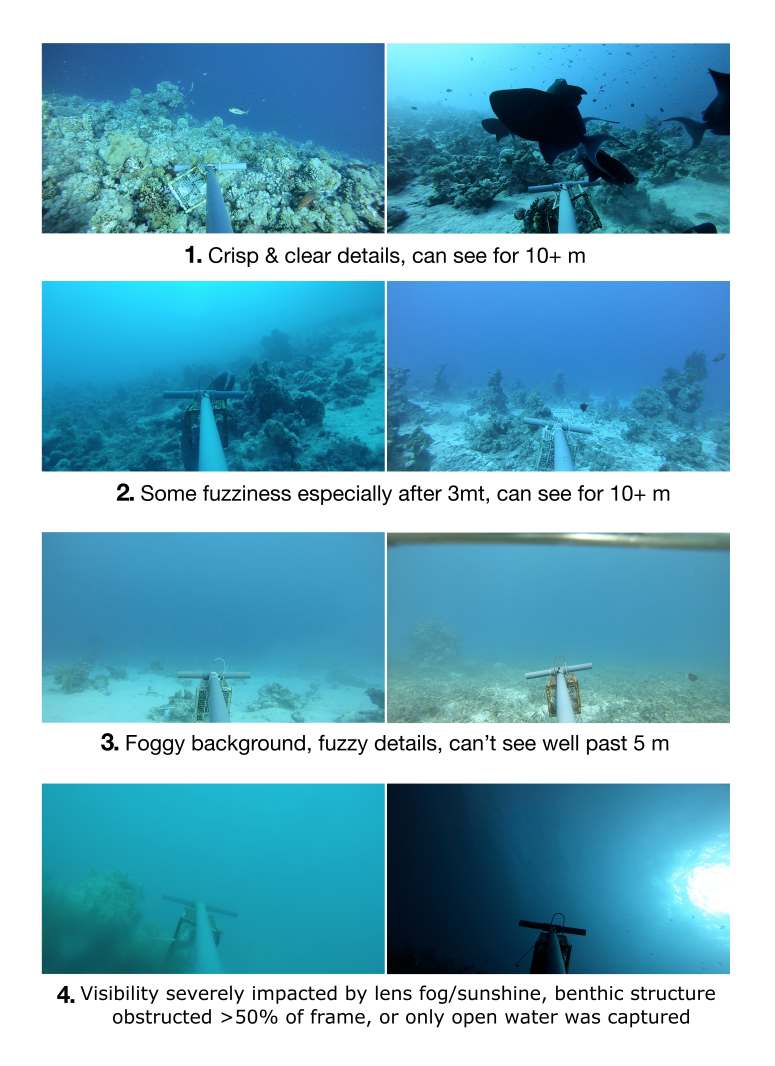


Figure S2: Habitat categories (H1-H3) used to describe environments surveyed with BRUVS in the Gulf of Aqaba and the northern Red Sea.


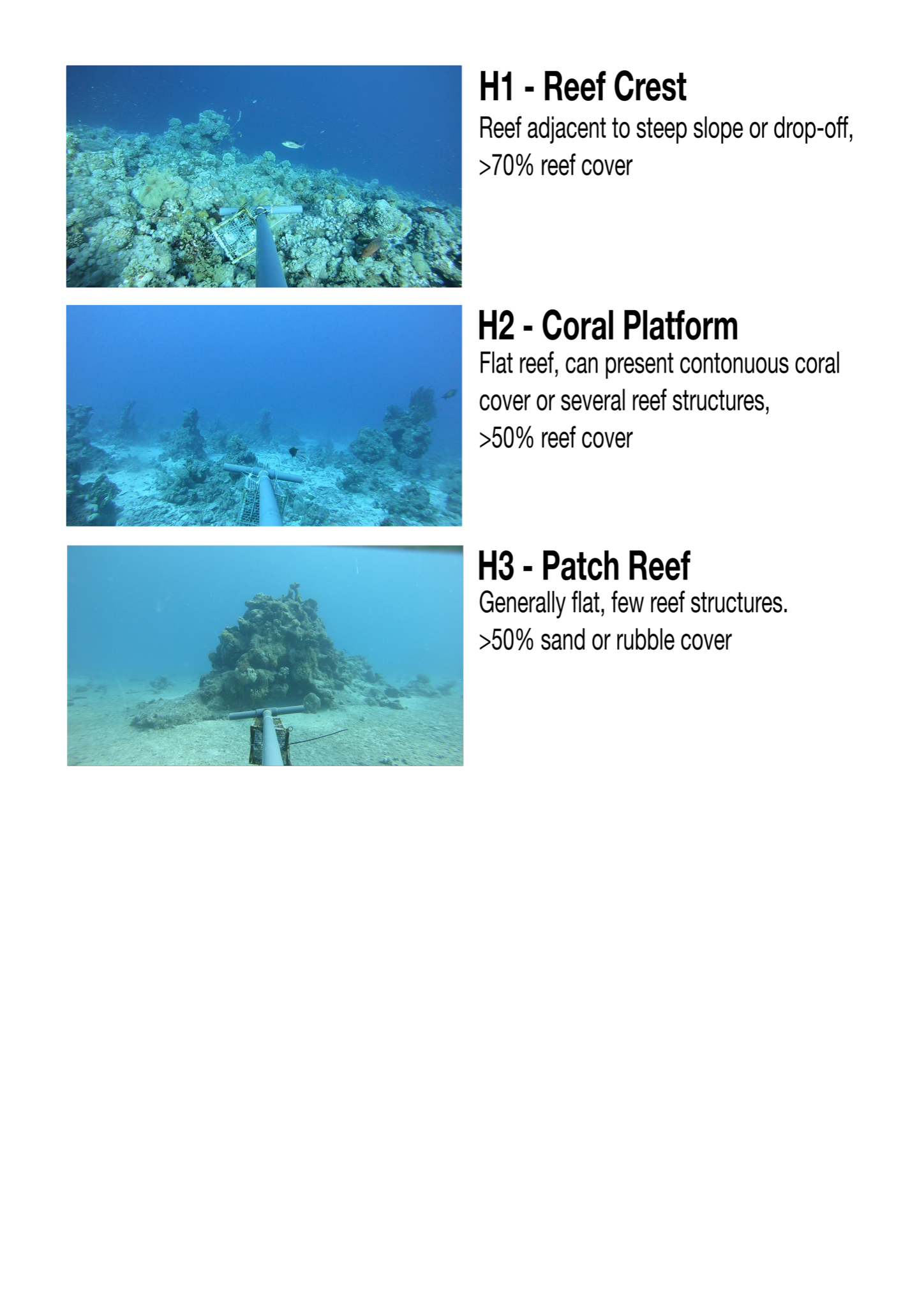


Figure S3: A coral reef in the Gulf of Aqaba near Haql, Saudi Arabia, covered in nylon monofilament fishing line (Photograph by Sean Ruggeri).


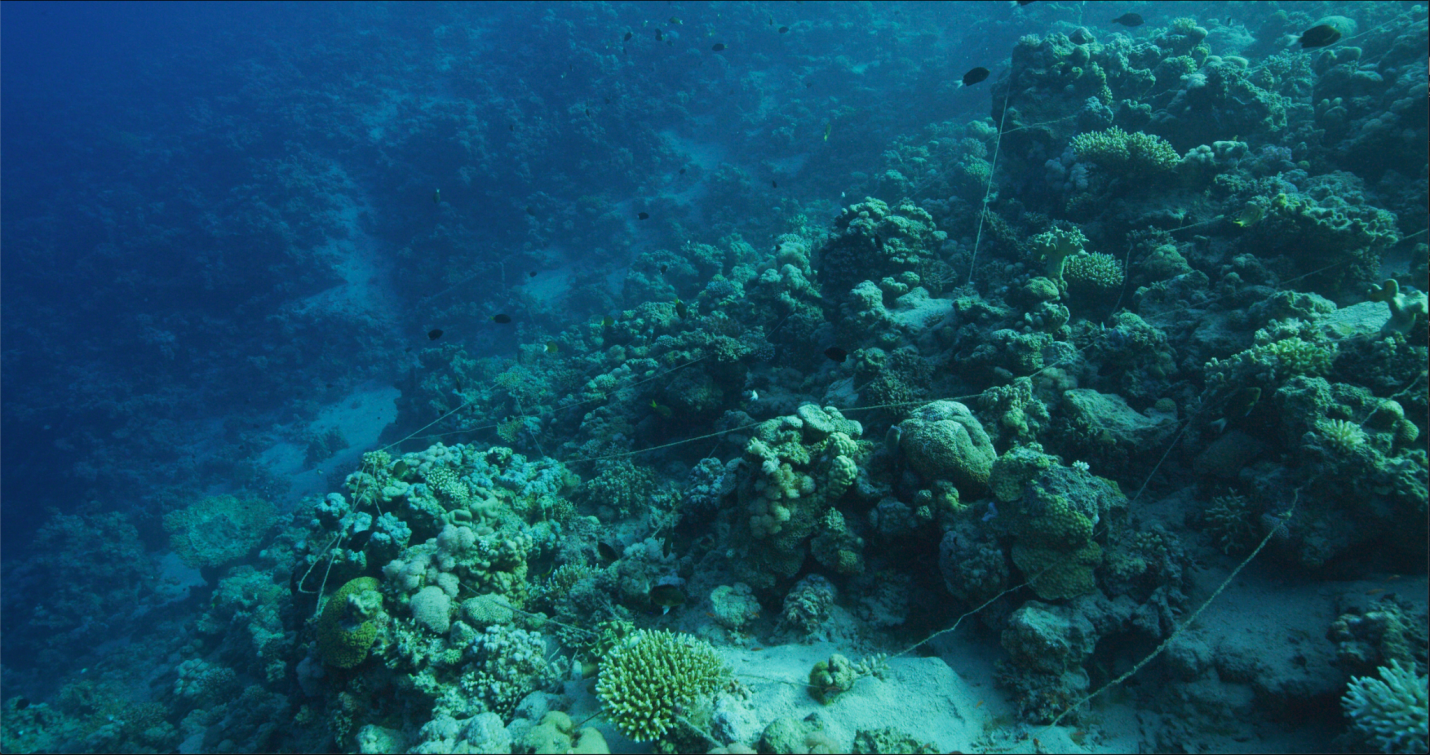


Table S1: Date and location of baited remote underwater video system (BRUVS) deployments in the Gulf of Aqaba (GOA) and northern Red Sea (NRS).

| **Deployment ID** | **Date** | **GOA/NRS** | **Lat** | **Lon** |
| --- | --- | --- | --- | --- |
| 001 | 2020-10-13 | NRS | 27.628517 | 35.318917 |
| 002 | 2020-10-13 | NRS | 27.628167 | 35.320050 |
| 004 | 2020-10-13 | NRS | 27.639900 | 35.306867 |
| 005 | 2020-10-14 | NRS | 27.596417 | 35.299600 |
| 006 | 2020-10-14 | NRS | 27.550850 | 35.481400 |
| 007 | 2020-10-17 | GOA | 29.121617 | 34.876267 |
| 008 | 2020-10-19 | GOA | 28.866400 | 34.838100 |
| 009 | 2020-10-20 | GOA | 28.802170 | 34.826850 |
| 012 | 2020-10-20 | GOA | 28.783510 | 34.819620 |
| 013 | 2020-10-20 | GOA | 28.780230 | 34.818540 |
| 014 | 2020-10-20 | GOA | 28.775330 | 34.814750 |
| 015 | 2020-10-20 | GOA | 28.768220 | 34.813900 |
| 016 | 2020-10-20 | GOA | 28.760560 | 34.812210 |
| 017 | 2020-10-21 | GOA | 29.219153 | 34.909246 |
| 018 | 2020-10-21 | GOA | 29.211825 | 34.910027 |
| 019 | 2020-10-21 | GOA | 29.206670 | 34.906331 |
| 020 | 2020-10-21 | GOA | 29.200309 | 34.904780 |
| 021 | 2020-10-22 | GOA | 29.201383 | 34.905400 |
| 022 | 2020-10-22 | GOA | 29.191550 | 34.899160 |
| 024 | 2020-10-22 | GOA | 29.178500 | 34.892930 |
| 025 | 2020-10-23 | GOA | 28.612180 | 34.795980 |
| 026 | 2020-10-23 | GOA | 28.606430 | 34.796250 |
| 027 | 2020-10-24 | GOA | 29.114160 | 34.874110 |
| 028 | 2020-10-24 | GOA | 29.103833 | 34.876167 |
| 029 | 2020-10-24 | GOA | 29.092217 | 34.867950 |
| 031 | 2020-10-24 | GOA | 29.041900 | 34.848950 |
| 032 | 2020-10-24 | GOA | 29.033367 | 34.846483 |
| 033 | 2020-10-24 | GOA | 29.026183 | 34.846150 |
| 034 | 2020-10-24 | GOA | 29.020283 | 34.852183 |
| 036 | 2020-10-24 | GOA | 28.952517 | 34.837983 |
| 037 | 2020-10-24 | GOA | 28.961050 | 34.839367 |
| 038 | 2020-10-24 | GOA | 28.970067 | 34.839050 |
| 039 | 2020-10-26 | GOA | 28.502640 | 34.791980 |
| 040 | 2020-10-26 | GOA | 28.494380 | 34.786260 |
| 041 | 2020-10-26 | GOA | 28.487870 | 34.778180 |
| 042 | 2020-10-26 | GOA | 28.478900 | 34.771770 |
| 043 | 2020-10-27 | GOA | 28.176200 | 34.630916 |
| 044 | 2020-10-27 | GOA | 28.171916 | 34.626830 |
| 045 | 2020-10-27 | GOA | 28.167683 | 34.623060 |
| 046 | 2020-10-27 | GOA | 28.164830 | 34.616783 |
| 047 | 2020-10-28 | NRS | 27.770520 | 35.146830 |
| 048 | 2020-10-28 | NRS | 27.776610 | 35.137680 |
| 050 | 2020-10-28 | NRS | 27.794200 | 35.123710 |
| 051 | 2020-10-28 | NRS | 27.773750 | 35.147390 |
| 052 | 2020-10-28 | NRS | 27.785630 | 35.142230 |
| 053 | 2020-10-28 | NRS | 27.788030 | 35.135810 |
| 054 | 2020-10-28 | NRS | 27.794000 | 35.125830 |
| 055 | 2020-10-28 | NRS | 27.775870 | 35.171000 |
| 056 | 2020-10-28 | NRS | 27.765410 | 35.184630 |
| 057 | 2020-10-28 | NRS | 27.762510 | 35.194550 |
| 058 | 2020-10-28 | NRS | 27.756230 | 35.208540 |
| 059 | 2020-10-29 | NRS | 27.783540 | 35.165210 |
| 060 | 2020-10-29 | NRS | 27.790140 | 35.163950 |
| 061 | 2020-10-29 | NRS | 27.788470 | 35.172940 |
| 062 | 2020-10-29 | NRS | 27.787610 | 35.184340 |
| 063 | 2020-10-30 | NRS | 27.644683 | 35.301967 |
| 064 | 2020-10-30 | NRS | 27.646967 | 35.293667 |
| 065 | 2020-10-30 | NRS | 27.655683 | 35.288750 |
| 067 | 2020-10-31 | NRS | 27.67747 | 35.253410 |
| 068 | 2020-10-31 | NRS | 27.67216 | 35.261150 |
| 069 | 2020-10-31 | NRS | 27.66655 | 35.266780 |
| 070 | 2020-10-31 | NRS | 27.65797 | 35.274400 |
| 071 | 2020-11-02 | NRS | 28.046910 | 34.723980 |
| 073 | 2020-11-02 | NRS | 28.037810 | 34.711340 |
| 074 | 2020-11-02 | NRS | 28.039110 | 34.705780 |
| 075 | 2020-11-02 | NRS | 28.043960 | 34.703730 |
| 076 | 2020-11-02 | NRS | 28.036970 | 34.702880 |
| 077 | 2020-11-02 | NRS | 28.029660 | 34.704200 |
| 078 | 2020-11-02 | NRS | 28.023050 | 34.697430 |
| 079 | 2020-11-03 | NRS | 28.07042 | 34.602280 |
| 081 | 2020-11-03 | NRS | 28.034150 | 34.606560 |
| 082 | 2020-11-03 | NRS | 28.020540 | 34.613090 |
| 083 | 2020-11-03 | NRS | 28.034540 | 34.581040 |
| 087 | 2020-11-04 | NRS | 27.901083 | 34.708783 |
| 088 | 2020-11-04 | NRS | 27.905183 | 34.69356 |
| 089 | 2020-11-04 | NRS | 27.931016 | 34.677183 |
| 090 | 2020-11-04 | NRS | 27.915930 | 34.682400 |
| 091 | 2020-11-04 | NRS | 27.943983 | 34.617033 |
| 092 | 2020-11-04 | NRS | 27.932283 | 34.626033 |
| 093 | 2020-11-04 | NRS | 27.915433 | 34.612667 |
| 094 | 2020-11-04 | NRS | 27.909633 | 34.596983 |
| 095 | 2020-11-05 | NRS | 27.955150 | 34.723430 |
| 096 | 2020-11-05 | NRS | 27.943400 | 34.733950 |
| 098 | 2020-11-05 | NRS | 27.929100 | 34.739110 |
| 099 | 2020-11-05 | NRS | 27.963010 | 34.690170 |
| 101 | 2020-11-06 | NRS | 27.942130 | 34.908440 |
| 102 | 2020-11-06 | NRS | 27.927350 | 34.898270 |
| 104 | 2020-11-06 | NRS | 27.950720 | 34.887850 |
| 105 | 2020-11-06 | NRS | 27.940760 | 34.872970 |
| 106 | 2020-11-06 | NRS | 27.944960 | 34.878840 |
| 107 | 2020-11-06 | NRS | 27.938050 | 34.866080 |
| 108 | 2020-11-06 | NRS | 27.932005 | 34.917179 |
| 109 | 2020-11-06 | NRS | 27.930582 | 34.909396 |
| 111 | 2020-11-07 | NRS | 28.020367 | 34.938483 |
| 112 | 2020-11-07 | NRS | 27.997817 | 34.949517 |
| 113 | 2020-11-07 | NRS | 27.995733 | 34.972533 |
| 114 | 2020-11-07 | NRS | 27.993250 | 34.960783 |
| 115 | 2020-11-07 | NRS | 28.007533 | 34.945000 |
| 116 | 2020-11-07 | NRS | 28.021850 | 34.962150 |
| 117 | 2020-11-08 | NRS | 28.073386 | 34.909530 |
| 118 | 2020-11-08 | NRS | 28.074515 | 34.898227 |
| 119 | 2020-11-08 | NRS | 28.075079 | 34.889325 |
| 120 | 2020-11-08 | NRS | 28.076061 | 34.879833 |
| 121 | 2020-11-08 | NRS | 28.073642 | 34.868851 |
| 122 | 2020-11-08 | NRS | 28.071182 | 34.858121 |
| 123 | 2020-11-08 | NRS | 28.069901 | 34.846031 |
| 125 | 2020-11-10 | NRS | 27.953430 | 34.503390 |
| 126 | 2020-11-10 | NRS | 27.963270 | 34.501960 |
| 127 | 2020-11-10 | NRS | 27.974960 | 34.497440 |
| 128 | 2020-11-10 | NRS | 27.984610 | 34.496460 |
| 131 | 2020-11-10 | NRS | 28.016240 | 34.486540 |
